# Different Kinds of Opium

**Published:** 1844-03-23

**Authors:** 


### DIFFERENT KINDS OF OPIUM.

According to Dr. O'Shaughnessy, Bengal investment opium (for the Chinese market) contains two and a half per cent. (by weight,) of pure morphia; Malwa opium, six per cent. ditto; Turkey opium, nine per cent. ditto; and garden opium (Patna) and Smyrna opium, ten and a half per cent. of morphia, each.

